# Enriched environmental exposure reduces the onset of action of the serotonin norepinephrin reuptake inhibitor venlafaxine through its effect on parvalbumin interneurons plasticity in mice

**DOI:** 10.1038/s41398-023-02519-x

**Published:** 2023-06-26

**Authors:** Basile Coutens, Camille Lejards, Guillaume Bouisset, Laure Verret, Claire Rampon, Bruno P. Guiard

**Affiliations:** Centre de Recherches sur la Cognition Animale (CRCA), Centre de Biologie Intégrative (CBI), CNRS UMR5169, Université de Toulouse, Toulouse, France

**Keywords:** Depression, Molecular neuroscience

## Abstract

Mood disorders are associated with hypothalamic-pituitary-adrenal axis overactivity resulting from a decreased inhibitory feedback exerted by the hippocampus on this brain structure. Growing evidence suggests that antidepressants would regulate hippocampal excitatory/inhibitory balance to restore an effective inhibition on this stress axis. While these pharmacological compounds produce beneficial clinical effects, they also have limitations including their long delay of action. Interestingly, non-pharmacological strategies such as environmental enrichment improve therapeutic outcome in depressed patients as in animal models of depression. However, whether exposure to enriched environment also reduces the delay of action of antidepressants remains unknown. We investigated this issue using the corticosterone-induced mouse model of depression, submitted to antidepressant treatment by venlafaxine, alone or in combination with enriched housing. We found that the anxio-depressive phenotype of male mice was improved after only two weeks of venlafaxine treatment when combined with enriched housing, which is six weeks earlier than mice treated with venlafaxine but housed in standard conditions. Furthermore, venlafaxine combined with exposure to enriched environment is associated with a reduction in the number of parvalbumin-positive neurons surrounded by perineuronal nets (PNN) in the mouse hippocampus. We then showed that the presence of PNN in depressed mice prevented their behavioral recovery, while pharmacological degradation of hippocampal PNN accelerated the antidepressant action of venlafaxine. Altogether, our data support the idea that non-pharmacological strategies can shorten the onset of action of antidepressants and further identifies PV interneurons as relevant actors of this effect.

## Introduction

Major depressive disorder (MDD) is characterized by a persistent low mood associated with other core symptoms, including suicidal ideation, loss of mental and physical energy, feelings of guilt, anhedonia, anxiety, and cognitive deficits [1]. The therapeutic activity of first-line treatments for MDD, such as the selective serotonin reuptake inhibitors (SSRIs) or the serotonin-norepinephrine reuptake inhibitors (SNRIs), relies on their ability to increase monoaminergic tone in the brain of depressed patients [2]. However, despite their indisputable efficacy, SSRIs and SNRIs suffer from several limitations including a delayed onset of action (4–8 weeks) [3], numerous adverse effects, and a modest efficacy in almost 30% of patients [4]. Although brain levels of serotonin (5-HT) and norepinephrine (NE) increase within hours after administration of an SSRI or SNRI, it is not fully understood why it takes weeks to obtain behavioral improvement. Current hypotheses to explain this delayed clinical response involve pre- and post-synaptic adaptive mechanisms. After an initial increase in intrasynaptic concentrations of neurotransmitters at the presynaptic level, SSRIs and SNRIs produce a progressive downregulation of somatodendritic inhibitory 5-HT_1A_ autoreceptors, increasing neuronal firing of 5-HT neurons and neurotransmitter release at nerve terminals, notably in the hippocampus [5]. Interestingly, the time required for antidepressant drugs to desensitize these 5-HT_1A_ autoreceptors coincides with their onset of action. Long-term changes in gene expression, protein translation, and neuroplasticity are also detected at the post-synaptic level. For instance, it is well known that increased hippocampal 5-HT and/or NE neurotransmissions in response to chronic administration of SSRIs or SNRIs promote BDNF expression [6], which positively regulates adult hippocampal neurogenesis [7] and parvalbumin (PV) GABAergic neuron maturation [8]. In addition, PV neurons also critically regulate adult hippocampal neurogenesis [9]. Hence, a better understanding of the mechanisms underlying the therapeutic action of antidepressant drugs would help to develop more efficient treatments, including faster-acting strategies.

Recent evidence suggests that the presence of perineuronal nets (PNN), an extracellular matrix located around fast-spiking GABAergic PV neurons, plays a role in mood regulation. PNN endows neuronal plasticity that could influence the behavioral response to antidepressant treatment [10, 11]. Accordingly, it has been shown in rodent models of depression, that chronic stress and corticosterone exposures increase hippocampal expression of PNN [12, 13]. On the contrary, the SSRI fluoxetine or the SNRI venlafaxine administered perinatally or in adulthood, decreases the formation of PNN around the soma and proximal dendrites of fast-spiking PV GABAergic interneurons in the mouse hippocampus [14–16] and cortex [17]. Furthermore, PNNs are dynamically regulated by experience, and exposure to an enriched environment results in a reduced presence of PNN around hippocampal PV cells [18, 19].

In the present study, we aimed to investigate whether the onset of action of the antidepressant venlafaxine can be shortened by combining this pharmacological treatment with exposure to an enriched environment. Then, to start identifying the cellular mechanisms underlying the shortened response to antidepressant, we focused on the extracellular matrix surrounding hippocampal PV interneurons.

## Materials and methods

### Animals

Ten-week-old male C57Bl/6Rj mice were housed five per cage under standard conditions with a 12 h light/dark cycle (light on at 8:00 a.m) and temperature-controlled room. Food and water were available *ad libitum*. All experimental procedures were conducted in accordance with the European directive 2010/63/EU and were approved by the French Ministry of Research and the local ethics committee (APAFIS # 2018100110245946#16913).

The enriched environment (EE) was performed in the Marlau™ cages (Viewpoint, France) which provides standardized environmental enrichment procedures for rodents. Living in Marlau cages increases social and sensory stimulation that evokes brain and cognitive reserves and supports functional rehabilitation after brain injury. The cage (length: 580 mm × width: 400 mm × height: 320 mm; weight: 13 kg) consists of a first floor with two compartments (one containing food, the other drinking water), and an upper floor where a maze is placed. To obtain food, the rodents must climb from the lower compartment to the upper floor, pass through the maze, and then descend to the other compartment through a sliding tunnel. Another pathway procedure provides access to drinking water. This ensures that all animals are frequently and equally exposed to the different features of the enrichment. According to the protocol for these cages, cognitive stimulation and curiosity are maintained over time through regular changes (three times a week) in the maze configuration, of which 12 different versions are available [20].

### Drugs administration

#### Corticosterone (CORT)

Mice were exposed to corticosterone (Sigma-Aldrich, France, Cat#C2505) dissolved in vehicle (0.45% β-cyclodextrin, Sigma, St Louis, MO) in drinking water for 8 weeks [21], and maintained during experimental procedures (35 μg/ml/day equivalent to 5 mg/kg/day as in [21]).

#### Venlafaxine (VLX)

During the last 8 weeks of corticosterone exposure, the SNRI venlafaxine (Sigma-Aldrich, France, Cat#99300-78-4) was dissolved in corticosterone solution and delivered in drinking water at a fixed dose of 16 mg/kg. This dose was chosen on the basis of our electrophysiological demonstration that it is the lowest dose allowing concomitant inactivation of SERT and NET and that this dose is effective to promote antidepressant effects after 3 weeks in CORT mice [22].

#### Doxycycline (DOX)

Inhibition of matrix metalloproteases was achieved by feeding mice a diet containing doxycycline. Doxycycline intake was about 5 mg/day per mouse (doxycycline hyclate, Ssniff, Germany).

#### Chondroitinase ABC (ChABC)

The chondroitinase ABC (ChABC) was used to degrade PNN. Mice were anesthetized with isoflurane (3%) (Centravet, France) and placed in a stereotaxic apparatus. Lidocaine was applied subcutaneously before surgery, then each mouse received two bilateral injections of 100nL of a solution containing ChABC (50 U/ml, Sigma-Aldrich, France) or vehicle (PBS 0.1 M) into the area CA1 of the dorsal hippocampus. The following coordinates were used (in mm from bregma): (AP) −1.34, (L) ± 1 and (V) −1.5 and (AP) −2.46, (L) ± 2 and (V) –1.5 mm. After recovery in a heated chamber, mice were returned to their home cages where they recovered for two days before behavioral testing.

### Behavioral tests

The same batteries of behavioral tests were performed for all the different experimental groups. These batteries encompassed six tests measuring emotional or cognitive aspects: the elevated plus maze (EPM), the tail suspension test (TST), the splash test, the novelty suppressed feeding test (NSF), the object location test (OL) and the three-chamber test. Description of these tests and parameters studied are described in Supplementary material.

#### Data analysis and z-scores

A z-score was calculated to integrate the performance of each animal across the comprehensive battery of behavioral tests. Details and rationale in animal behavior analysis have been described by Guilloux et al. [23]. The z-score is used to compare overall behavioral performance between the experimental and control groups. Briefly, for each behavioral measure, an individual Z-score is calculated using the following equation:

$${{{\mathrm{Z}}}} = \left[ {\left( {{{{\mathrm{X}}}} - \mu } \right)/\sigma } \right]$$

X represents the individual data for the observed parameter. µ and σ represent the mean and standard deviation for the control group, respectively. Z-score indicates how many standard deviations (SD) observations (X) are above or below the mean of the control group (µ).

**Fig. 1:**
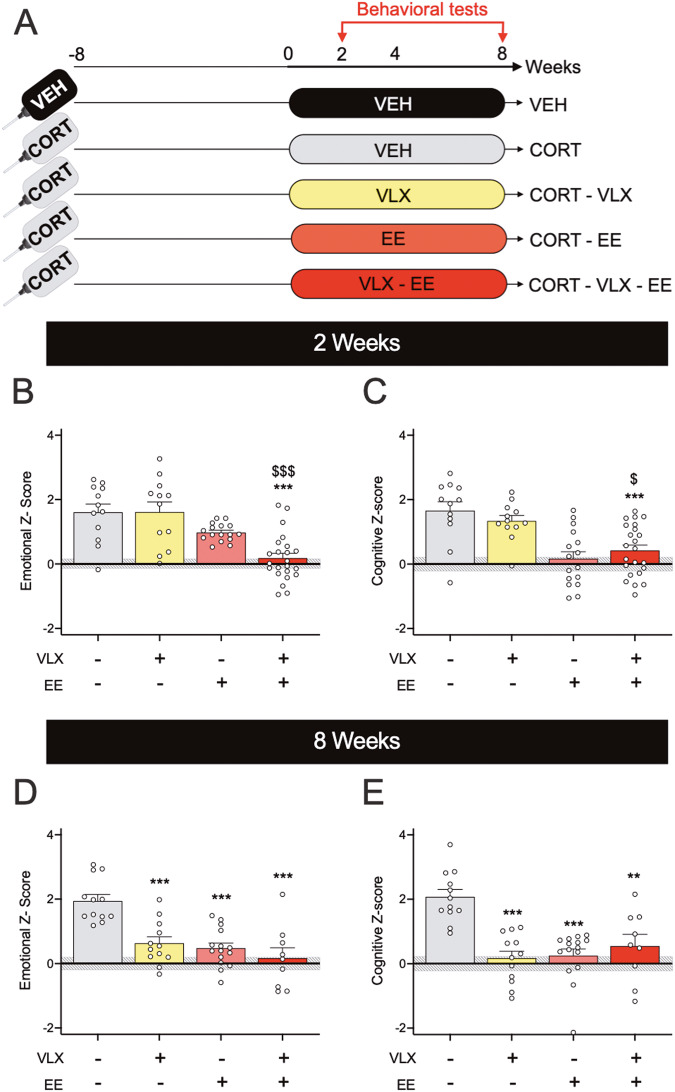
Exposure to enriched environment shortens the time to antidepressant action of venlafaxine. **A** Experimental timeline. Animals were exposed to corticosterone (CORT mice, *n* = 63) or vehicle (VEH mice, *n* = 12) in the drinking water during the whole experiment. Starting at week 0, CORT mice received a treatment of venlafaxine or vehicle for 8 weeks either in their home cage (CORT-VLX mice, *n* = 12 and CORT-VEH mice, *n* = 12) or in an enriched environment (CORT-EE mice, *n* = 16 and CORT-EE-VLX mice, *n* = 23). Behavior was evaluated after 2 (**B**, **C**) and 8 (**D**, **E**) weeks of treatment. **B**, **D** Emotional and (**C**, **E**) cognitive tests were analyzed using two-way ANOVAs with housing condition and treatment as main factors. Emotional and cognitive z-scores were established based on VEH mice. After 2 weeks of treatment, a significant effect of housing condition (*F*_(1;59)_ = 27.0, *p* < 0.001) and housing condition × treatment interaction (*F*_(1;59)_ = 4.16, *p* < 0.05) were found for emotional z-score. A significant effect of housing condition was detected for cognitive z-score (*F*_(1;59)_ = 32.0, *p* < 0.001). After 8 weeks of treatment, ANOVA revealed a significant effect of housing condition, treatment and housing condition × treatment interaction for the emotional (*F*_(1;44)_ = 21.0, *p* < 0.001, *F*_(1;44)_ = 14.9, *p* < 0.001, and *F*_(1;44)_ = 5.57, *p* < 0.05, respectively) and the cognitive (*F*_(1;44)_ = 8.39, *p* < 0.01, *F*_(1;44)_ = 10.4, *p* < 0.01, and *F*_(1;44)_ = 19.5, *p* < 0.001, respectively) z-scores. The gray shaded area shows mean ± SEM values for VEH mice. In all other groups, data represent mean ± SEM and dots illustrate individual values. Post-hoc analysis when appropriate: **p < 0.01, ***p < 0.001 indicate significant differences compared to CORT-VEH group. ^$^*p* < 0.05, ^$$$^*p* < 0.001 indicate significant differences compared to CORT-VLX group.

**Fig. 2:**
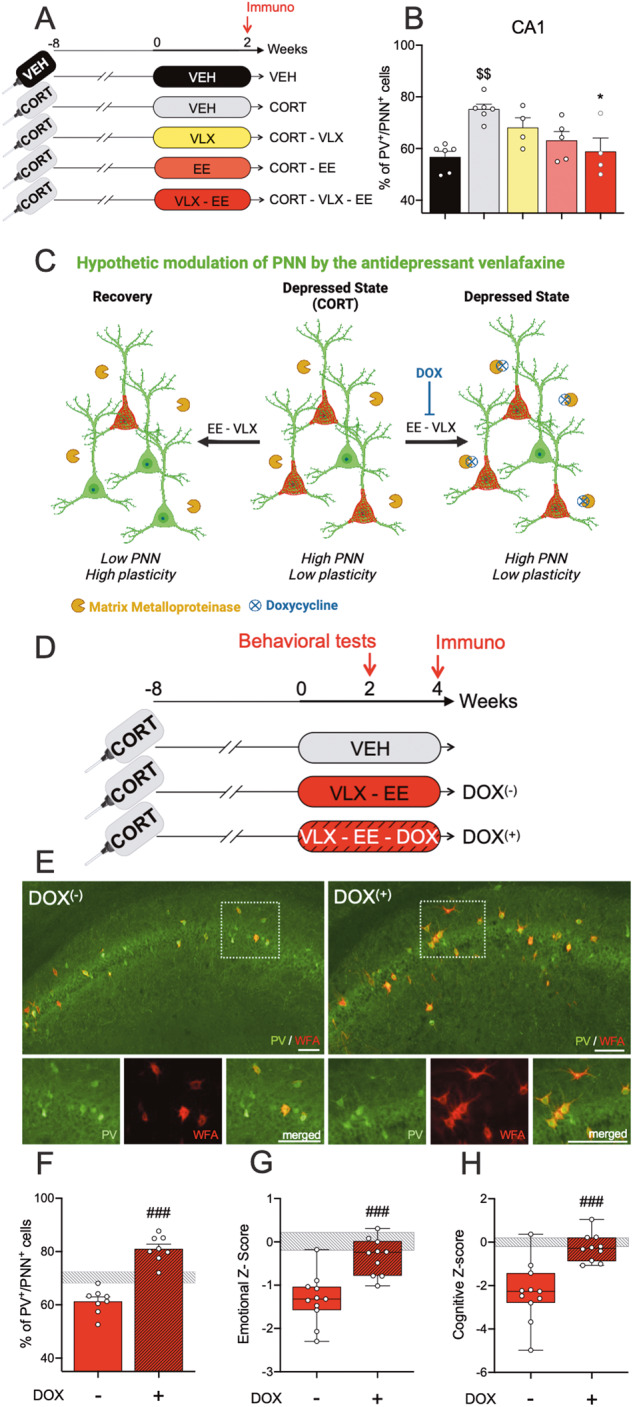
The antidepressant action of VLX requires remodeling of the extracellular matrix surrounding hippocampal interneurons. **A** Experimental timeline. All along the experiment, animals were exposed to corticosterone (CORT mice, *n* = 19) or vehicle (VEH mice, *n* = 6) in the drinking water. From week 0, CORT mice received venlafaxine or vehicle for 2 weeks either in their home cage (CORT-VLX mice, *n* = 4 and CORT-VEH mice, *n* = 6) or in an enriched environment (CORT-EE mice, *n* = 5 and CORT-EE-VLX mice, *n* = 4). Animals were sacrificed after 2 weeks of treatment and brain were processed for histology. **B** Proportion of PV^+^/PNN^+^ cells among PV^+^ cells in CA1. One-way ANOVA analysis reveals significant difference (*F*_(4;20)_ = 6.378, *p* < 0.01). **C** Scheme representing the hypothetic modulation of PNN (in red) by venlafaxine (VLX) and enriched environment (EE) in the hippocampus. In depressed state (CORT), we observed that the number of PV^+^ cells surrounded by PNN increases compared to basal state (VEH) while the combination VLX and EE abolishes this effect, allowing neuronal plasticity and behavioral recovery. In contrast, doxycycline (DOX) inhibits matrix metalloproteases known to degrade PNN. This enables the stabilization of PNN networks around PV^+^ cells and prevents neuronal plasticity. **D** Experimental protocol used to study the impact of the pharmacological blockade of PNN remodeling. Starting at week 0, CORT mice (*n* = 29) received venlafaxine for 2 weeks in EE. Mice were either fed a normal diet (CORT-VLX-EE-DOX^(−)^, *n* = 11) or a diet containing DOX (CORT-VLX-EE-DOX^(+)^, *n* = 10). Emotional and cognitive z-scores were established from mice exposed to CORT alone (CORT-VEH mice, *n* = 8). **E** Photomicrographs depicting PV^+^/PNN^+^ cells in the CA1 region of CORT-depressed mice housed EE and treated with VLX (VLX-EE) or with the combination of VLX and DOX (VLX-EE-DOX). **F** Proportion of PV^+^/PNN^+^ cells among PV^+^ cells in CA1. A significant increase is observed in DOX fed mice (unpaired Student *t*-test). **G** Emotional and (**H**) cognitive z-scores showing a significant effect of DOX using unpaired Student *t*-test. The gray shaded area shows mean ± SEM values for CORT mice. Data represent mean ± SEM and dots illustrate individual values. Post-hoc and *t*-test analysis when appropriate: ^*^p < 0.05 indicates significant differences compared to CORT-VEH group. ^$$^*p* < 0.01 indicates significant differences compared to VEH group. ^###^*p* < 0.001 indicates significant differences between groups. Scale bars = 50 µm.

**Fig. 3:**
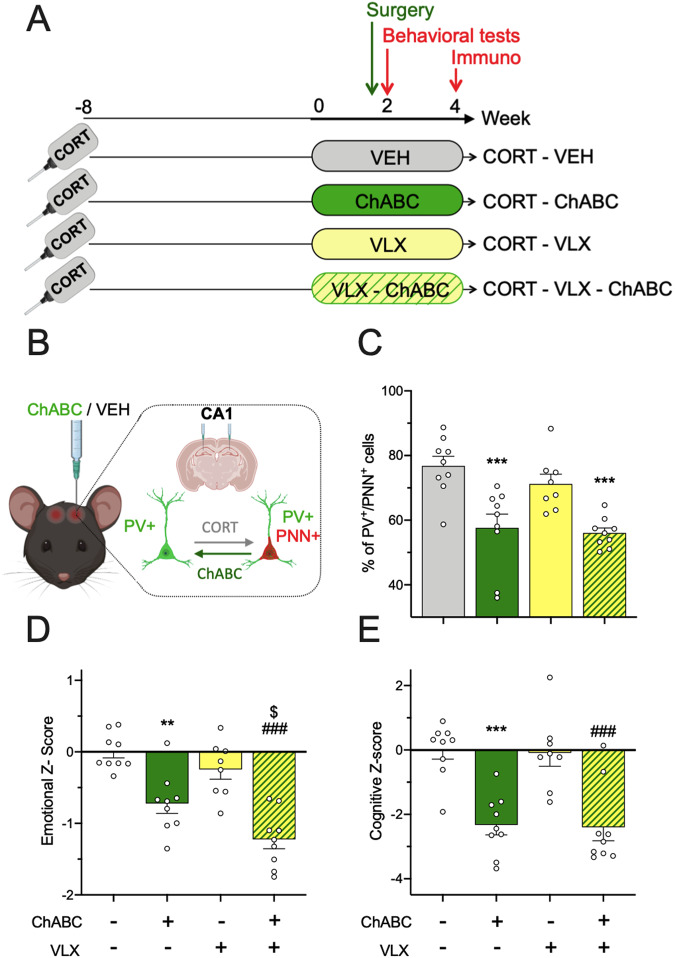
Pharmacological degradation of hippocampal PNN replicates the rapid antidepressant action of VLX + EE treatment. **A**, **B** Experimental timeline. Animals were exposed to corticosterone (CORT mice, *n* = 35) in the drinking water throughout the experiment. From week 0, CORT mice were received venlafaxine or vehicle for 2 weeks in their home cage (CORT-VEH mice, *n* = 9 and CORT-VLX mice, *n* = 8). These groups were divided into two additional groups receiving intra-hippocampal injections of the PNN degrading enzyme ChABC, or vehicle. (CORT-VEH mice: *n* = 9, CORT-VLX mice: *n* = 8, CORT-ChABC mice: *n* = 9; and CORT-VLX-ChABC mice: *n* = 9). **C** Proportion of PV+/PNN+ cells among PV+ cells in CA1. One-way ANOVA with treatment as main factor revealed a significant effect of treatment (*F*_(3;31)_ = 11.00, *p* < 0.001). Emotional (**D**) and cognitive (**E**) z-scores were calculated and a two-way ANOVA with pre-treatment (VLX vs. VEH) and treatment (ChABC vs. vehicle) factors was applied. For the emotional z-score, a significant effect of pre-treatment (VLX vs. VEH) and treatment factors (ChABC vs. VEH) but not of their interaction was unveiled (*F*_(1;31)_ = 9.06, *p* < 0.01, *F*_(1;31)_ = 47.0, *p* < 0.001, *F*_(1;31)_ = 1.08, *p* = 0.301, respectively). For the cognitive z-score (**E**), a significant effect of treatment but not of pre-treatment nor their interaction was unveiled (*F*_(1;31)_ = 41.7, *p* < 0.001, *F*_(1;31)_ = 0.05, *p* = 0.824, *F*_(1;31)_ = 0.00, *p* = 0.985, respectively). Data represent mean ± SEM, dots illustrate individual values. Post-hoc analysis when appropriate: **p < 0.01, ***p < 0.001 indicate significant difference compared to CORT-VEH group. ^$^*p* < 0.05 indicates significant difference compared to CORT-ChABC group. ^###^*p* < 0.001 indicates significant difference compared to CORT-VLX group.

Two separate z-scores were established to account for the emotional and cognitive aspects of the behavioral measures. In these z-scores, each test is weighted equally. The data integrated in the emotional z-score includes the following parameters: percentage of time spent in open arms for the EPM, latency to feed for NSF, immobility time in the TST, time of grooming in the splash test. The data integrated in the cognitive z-score includes social novelty during the three-chamber test and preference for the displaced object in the object location test. For the experiments shown in Figs. 1 and 2, the control group consisted of the VEH animals. Thus, an increase of Z-scores indicates a deterioration in emotional and cognitive performance compared to the VEH group. For the experiment shown in Fig. 3, the control group is composed of CORT animals, so the reduced z-scores represent improved emotional and cognitive performance compared to the CORT group.

### Stereotaxic injection

For PNN degradation by the chondroitinase ABC (ChABC), mice were anesthetized with isoflurane (3%) and placed in a stereotaxic apparatus (Kopf). Lidocaine was applied subcutaneously before surgery, then each mouse received two bilateral injections of 100 nL of a solution containing ChABC (50 U/ml, Sigma-Aldrich) or vehicle (PBS 1×) into the area CA1 of the dorsal hippocampus. The following coordinates were used (in mm from bregma): (AP) −1.34, (L) ± 1 and (V) −1.5 and (AP) −2.46, (L) ± 2 and (V) –1.5 mm. After recovery in a heated chamber, mice were returned to their home cages where they recovered for two days before behavioral testing.

### Brain preparation

At the end of behavioral experiments, mice were deeply anesthetized with dolethal and perfused with NaCl at room temperature for 1 min while cold NaCl (4 °C) was used in the experiment involving doxycycline. Then brains were harvested and dissected into right and left hemispheres. For immunochemistry, hemi-brains were fixed in 4% paraformaldehyde solution for 48 h at 4 °C and then stored in 30% sucrose solution with 0.1% sodium azide. For protein quantification by ELISA, hippocampi were dissected from the remaining hemi-brains quickly frozen in liquid nitrogen.

### ELISA of MMP9

Homogenates from hippocampal tissue were prepared by lysis in immunoprecipitated buffer (RIPA - Thermo Scientific, 89901) with protease inhibitor cocktail (P8340-1ML, Sigma-Aldrich). Lysates were sonicated for 10 s, placed on ice for 20 min, and centrifuged 15 min at 14,000 rpm at 4 °C. Lysate supernatants were saved for protein analyses. Pro-MMP9 protein concentration in hippocampal lysates was measured by ELISA, performed according to the manufacturer’s protocol (Mouse Pro-MMP9, R&D systems, catalogue number MMP900B).

### Immunochemistry of PV and PNN

Floating coronal sections (30 µm thick) were prepared with a freezing-stage microtome (Leica SM2010R) and stored in cryoprotectant at −20 °C until use. For each animal, series of 1-in-6 sections spanning the hippocampus were washed in PBS + 0.25% Triton-X-100 (PBST). Then sections were placed in 3% H_2_O_2_ and 10% methanol in PBST and washed again in PBST before incubation in blocking solution for one hour (PBST containing 10% normal donkey serum). Finally, sections were incubated in the blocking solution containing biotinylated Wisteria Floribunda Agglutinin (WFA); Sigma L1516; 1:1000) and Goat anti-PV (Swant PVG 213; 1:2500) antibodies, overnight at room temperature. The next day, sections were rinsed in PBST and incubated for 90 min at room temperature with Donkey anti-Goat Alexa 488 (Molecular Probes, A11055; 1:250) and Streptavidin TRITC (Vector Labs, SA-5549, 1:500). Then sections were mounted onto slides, coverslipped using Mowiol containing Hoechst (1/10,000), and stored at 4 °C. Examination of positively labeled cells was confined to the dorsal hippocampus CA1. Quantifications of PV-immunoreactive (PV^+^) cells and PV-PNN immunoreactive (PV^+^/PNN^+^) cells were conducted using a DM6000B fluorescence microscope (Leica, Germany) equipped with a motorized X–Y sensitive stage and a video camera connected to a computerized image analysis system (ExploraNova, France).

### Quantification of PV^+^ cells and their associated PNN

The counting of labeled cells was conducted using Mercator v.2 software (ExploraNova) to measure the corresponding hippocampal surface. Densities of immuno-positive cells were calculated by dividing the number of positive cells by the region of interest (ROI) sectional volume. The densities of PV^+^ cells and PV^+^ cells enwrapped by PNN (PV^+^/PNN^+^) were calculated for each section, to obtain the percentage of colocalization of PV^+^ co-expressing PNN+ around them for each ROI.

### Randomization and blinding

Given that treatments were given in the drinking water (e.g., CORT and/or venlafaxine) in standard or enriched environments, randomization was not possible in the majority of the experiments. However, randomization was applied for the pharmacological experiments involving the intra-hippocampal injection of ChABC or its vehicle. In the latter experiments, animals receiving the different treatment were mixed in the same cage. With respect to the blinding, the experimentors remained blind to the experimental conditions until the end of data analysis.

### Statistical analysis

All data were expressed as mean ± standard error of the mean (SEM) unless stated otherwise. Analyses were performed using GraphPad Prism 8 (GraphPad Software, San Diego, CA, United States) and compared by t-test, one-way or two-way analysis of variance (ANOVA) followed by Tukey’s post hoc tests as mentioned in the figure legends. The analyzing statistic difference was indicated in the figure legends. The statistical power and the required sample size were based on our previous analysis using the same tests. No animals were excluded from the study and the number of animals per group can be found in the statistics tables (see supplemental data) as well as on the graphs with the dots representing the individual values. Null hypothesis was rejected when *P* < 0.05.

## Results

### Long term exposure to CORT induces a depressive-like phenotype in mice

We used mice chronically exposed to CORT as an animal model of depression. Deploying a comprehensive battery of tests to address emotional and cognitive symptoms of depression, we confirmed that CORT-exposed mice display a depressive-like phenotype. These symptoms included anxiety, resignation, impairments in self-care, spatial memory and social recognition compared to non-depressed control mice (Supplemental Fig. S1 and Supplemental Table S1). Altogether these findings are in line with previous reports and support that chronic exposure to CORT elicits a robust depressive-like phenotype in mice [21, 24].

### Long-term treatment with venlafaxine is necessary to induce antidepressant effects

We next sought to determine the impact of the duration of treatment with the antidepressant venlafaxine (VLX) on the CORT mouse model of depression. To do so, we evaluated the behavioral effects of a short (2 weeks) or a long (8 weeks) period of antidepressant treatment (Fig. S1).

After 2 weeks of venlafaxine, the performances of CORT-VLX mice did not differ from those of CORT mice, in any of the emotional or cognitive tests (Fig. S1A, B, C, E, G, H, I and K). Specifically, venlafaxine had no effect on anxiety evaluated by the time spent in the anxiogenic open arms of the elevated plus-maze (Fig. S1A). Venlafaxine also did not reverse the action of CORT in the novelty suppressed feeding test assessing hyponeophagia, another symptom of anxiety (Fig. S1C). Furthermore, venlafaxine does not show any effect on self-care assessed by the time of grooming in the splash test (Fig. S1E). Resignation was evaluated in the tail suspension test, and 2 weeks of venlafaxine failed to reverse the depressive traits of CORT mice, as reflected by a high immobility (Fig. S1G). At the cognitive level, deficits of the CORT mice were also not improved by 2 weeks of venlafaxine, as shown for spatial memory in the object location test (Fig. S1I) and for social recognition in the three-chamber test (Fig. S1K). Overall, 2-week venlafaxine treatment does not abolish the depressive-like symptoms in this mouse model of depression, indicating that a longer treatment with venlafaxine is necessary to elicit antidepressant-like effects [25].

To test this idea, behavioral performances of CORT-VLX mice were assess after 8 weeks of venlafaxine. We found that this long-term treatment abolished anxiety (Fig. S1B), hyponeophagia (Fig. S1D), resignation and self-care (Fig. S1F, H) symptoms of depressed mice. Moreover, 8 weeks of venlafaxine also restored spatial (Fig. S1J) and social (Fig. S1L) memory of depressed mice to control levels.

Altogether, our data indicate that antidepressant-like effects of venlafaxine are observed after 8, but not 2, weeks of treatment.

### Exposure to enriched environment greatly shortens the time to antidepressant action of venlafaxine

In an attempt to solve this issue, we investigated whether combining venlafaxine with non-pharmacological approaches would shorten the onset of action of venlafaxine in the CORT mouse model of depression. Indeed, cognitive and physical stimulations provided by housing mice in enriched environment (EE) have long been shown to exert a positive impact on behavior and brain plasticity [26].

We thus evaluated the impact of 2 and 8 weeks of venlafaxine and EE, alone or in combination, on depressive symptoms of CORT mice (Fig. 1A). Using the same battery of behavioral tasks as in Fig. S1, emotional and cognitive z-scores that integrated all related parameters into a single value (see methods) were calculated for each mouse and standardized to the values of non-depressed control mice.

Housing condition and the interaction between these two factors revealed statistical differences (Fig. 1B, Table S2). After 2 weeks of treatment, post-hoc analysis revealed that the combination of VLX with EE significantly decreased the emotional z-score of CORT mice while each strategy applied separately failed to do so. At the cognitive level, only the housing condition factor showed a significant effect. While 2 weeks of venlafaxine alone failed to improve the cognitive z-score of depressed mice, EE reduced the cognitive deficits observed in CORT-depressed mice treated or not with venlafaxine (Fig. 1C).

After 8 weeks of venlafaxine, a significant effect of both factors (treatment and housing condition) but also their interaction was unveiled for the behavioral z-scores (Fig. 1D, E). Emotional and cognitive impairments of depressed mice were overcome by VLX, EE alone or their combination. It is noteworthy that all three strategies (VLX, EE, their combination) were equally effective in decreasing emotional and cognitive z-scores, with no statistical difference found between them.

Together our data demonstrate that exposure to EE shortens the response time to venlafaxine by 8 to 2 weeks, particularly with regards to emotional alterations associated with depression. The neurobiological substrate underlying these beneficial effects remains to be identified.

### The antidepressant action requires the remodeling of the extracellular matrix surrounding hippocampal interneurons

Growing evidence suggests that the extracellular matrix PNN enwrapping PV cells participates to antidepressants response by reinstating hippocampal plasticity (for review [27]). We thus sought to determine whether manipulating this form of neuronal plasticity could influence the delay of action of venlafaxine in the CORT mouse model of depression.

First, we examined whether PNN presence was affected by 2 weeks of venlafaxine treatment, alone or combined with EE, in the CORT mouse model of depression (Fig. 2A). We found that CORT exposure enhanced the percentage of PV-labeled (PV^+^) cells enveloped by PNN (PV^+^/PNN^+^ cells) in the dorsal part of the CA1 hippocampal region (Fig. 2B, Table S3), while the density of PV^+^ interneurons did not vary in CA1 (Fig. S2). Of all treatments, only the 2 weeks combination of VLX and EE significantly reduced the proportion of PV^+^/PNN^+^ cells in CA1, compared to depressed mice (Fig. 2B). Although venlafaxine or EE alone tended to lower the proportion of PV^+^ interneurons enveloped by PNN, these effects did not reach significance (Fig. 2B).

Work by Kwok et al. [28] suggests that PNN around neurons prevents the formation of new synapses on PV interneurons, and thereby participates in hippocampal plasticity. In this context, our data suggest that the combination of VLX and EE, by allowing the remodeling of PV-dependent network, could contribute to the behavioral recovery of depressed mice.

To investigate this possibility, we asked whether the plasticity mediated by the presence of PNN around PV^+^ neurons is required for the rapid antidepressant action of venlafaxine combined with EE, in the CORT mouse model. We hypothesized that preventing the degradation of PNN around PV^+^ interneurons in depressed mice treated with VLX and exposed to EE would hinder hippocampal plasticity and block the antidepressant action of this combined treatment. To prevent PNN degradation, concomitantly with venlafaxine administration, animals housed in EE were fed a diet containing doxycycline, an inhibitor of matrix metalloprotease 9 (MMP9), the PNN degradation enzyme [29, 30] (Fig. 2C, D).

According to this idea, we observed that doxycycline reduces hippocampal expression of MMP9 using ELISA quantification, although this effect remained below significance (Fig. S3). Immunohistological analysis of the CA1 region confirmed that the proportion of PV^+^ cells harboring PNN in mice receiving the VLX + EE combination was similar to the proportion observed in the previous experiment (around 60%, Fig. 2A, B vs. Fig. 2E, F). Remarkably, and confirming our hypothesis, we observed that the action of VLX + EE on PV^+^/PNN^+^ cell numbers was blocked when PNN degradation was prevented by doxycycline (Fig. 2E, F). Altogether, these findings suggest that in CORT-depressed mice, VLX + EE treatment reduces PNN expression around PV^+^ cells, allowing experience-dependent remodeling within the hippocampus.

We then evaluated the behavioral effects of VLX + EE treatment in CORT depressed mice fed a doxycycline-containing diet. In these mice, emotional and cognitive z-scores remained robustly impaired compared to animals fed with a standard diet (Fig. 2G, H). These findings demonstrate that inhibiting PNN degradation with doxycycline prevents behavioral recovery in depressed mice. Collectively, these data show that restricting the synaptic plasticity onto hippocampal PV^+^ interneurons blocks the rapid antidepressant action of VLX + EE combination. They also further suggest that rapid antidepressant action of VLX + EE tightly depends on the regulation of the extracellular matrix around hippocampal PV neurons and/or of the subsequent PV cell activity.

### Pharmacological degradation of hippocampal PNN replicates the rapid antidepressant action of VLX + EE treatment

To assess to which extent, hippocampal PNN remodeling is crucial to the rapid action of VLX + EE, we performed intra-hippocampal administrations of the chondroitinase ABC (ChABC), a bacterial enzyme that degrades PNN [31]. After only 2 weeks of VLX treatment and just before behavioral testing, ChABC was infused into CORT-depressed mice CA1 region (Fig. 3A, B).

As expected, ChABC massively degraded the PNN at the injection sites, resulting in a significant reduction in the proportion of PV^+^ neurons embedded in PNN, compared to depressed mice receiving vehicle (Fig. 3C, Table S4). Of note, the combination of ChABC and venlafaxine treatment did not produce a more pronounced effect on the proportion of PV^+^/PNN^+^ neurons in CA1 than ChABC alone (Fig. 3C). At the behavioral level, emotional and cognitive z-scores were calculated for each mouse and normalized to the values of CORT-depressed control mice that received intra-hippocampal injection of vehicle. While emotional z-score showed a significant effect of the two main factors (pre-treatment and treatment), no significant effect of their interaction was unveiled. With respect to the cognitive z-score, only a significant effect of treatment was detected. Based on these analyses, we found that ChABC induced an early antidepressant-like effect in the absence of venlafaxine treatment, as evidenced by significantly lower emotional and cognitive z-scores of CORT-ChABC mice compared to CORT-VEH animals (Fig. 3D, E). The same statistical observations were found among venlafaxine-treated mice. Remarkably, among ChABC-injected mice, venlafaxine improved the emotional state of depressed mice (Fig. 3D). These results strongly suggest that the restoration of PV cell remodeling, mediated by the absence of PNN is one of the neurobiological mechanisms by which EE shortens the time to antidepressant action of venlafaxine.

Collectively our results demonstrate that venlafaxine antidepressant action is faster when combined with exposure to a stimulating environment. Furthermore, they reveal that regulation of the hippocampal extracellular matrix could be one of the molecular actors involved in the delayed onset of action of this antidepressant drug.

## Discussion

Our study shows that the antidepressant-like effects of the SNRI venlafaxine in depressed mice are achieved earlier when this compound is combined with animal’s exposure to an enriched environment (EE) providing social, physical, and cognitive stimulations. We propose that the remodeling of the extracellular matrix PNN located around hippocampal parvalbumin interneurons may be a pivotal cellular mechanism underlying the rapid antidepressant effects of venlafaxine combined with EE. Indeed, we have gathered evidence that maintaining hippocampal PNN’s integrity hinders the antidepressant-like effects of venlafaxine and EE combination; in contrast, disrupting hippocampal PNN allows rapid beneficial effects on emotional and cognitive hallmarks of the depressive state.

The chronic mouse model of CORT exposure was used to induce a robust and persistent depressive-like phenotype associated with cognitive deficits. One limitation of this model is that C57BL6J female mice are insensitive to long term administration of corticosterone [32], which led us to test the impact of different antidepressant strategies on the time course of behavioral recovery on males only. Using emotional and cognitive z-scores capturing the heterogeneity of depressive-symptoms [23], we show that venlafaxine at the lowest dose (16 mg/kg) to enhance serotonergic and noradrenergic neurotransmissions in mice [22], abolishes CORT-induced behavioral deficits after 8 weeks of treatment. In these animals, venlafaxine has, however, no effect after only 2 weeks. The dose used herein is an important factor in this lack of effect as it has been reported that 30 mg/kg of venlafaxine was sufficient to induce antidepressant-like effects after 2 weeks of treatment [13]. However, our results are consistent with previous data showing that 3–5 weeks are required after initiation of the treatment with the SSRI fluoxetine or SNRI venlafaxine to elicit improvement in behavioral parameters [22, 33, 34]. Mechanistically, this delay of action of antidepressant drugs coincides with the desensitization of the inhibitory somatodendritic 5-HT1A autoreceptors and the enhancement of hippocampal neurotransmission that occurs unambiguously after more than 2 weeks of venlafaxine administration [35]. Although non-pharmacological interventions such as exposure to EE were shown to exert antidepressant-like effects [36], their ability to improve behavioral response to antidepressant drugs has been much less studied. A study in rats reported that the SSRI sertraline exerts anxiolytic-like effects when administered during EE but not in standard or isolated housing conditions [37]. Similar findings were reported in different mouse or rat models of depression using the combination of the SSRI fluoxetine and EE [38–41]. Consistent with these findings, our results show that 2 weeks of venlafaxine administration combined with EE exposure produces beneficial effects on the emotional z-scores of depressed mice, whereas venlafaxine or EE applied separately failed to do so. Also in line with a previous report, the effect of EE alone on cognitive z-score is not enhanced when combined with venlafaxine, likely due to its own robust impact on this parameter [42].

PNN are a complex of extracellular matrix molecules that mostly surround the soma and dendrites of fast-spiking GABAergic neurons in various brain regions. PNN are functionally involved in the stabilization of excitatory synapses onto PV cells [43] and they have been reported to play a crucial role in hippocampal plasticity and thus, in memory processes [44]. Emerging evidence suggests that PNN also control stress response and emotional state. Specifically, increased PNN formation is observed in the hippocampus of rodents chronically exposed to CORT [13] or to social defeat [12]. Such an increase may contribute to reduce hippocampal plasticity and to impair emotional and cognitive abilities of stressed mice [45–47]. In agreement with this hypothesis, it has been shown that a 2-week administration of the SNRI venlafaxine reduces PNN immunoreactivity in mice hippocampus [15]. Other studies have demonstrated that chronic fluoxetine treatment also reduces the density of PNNs and PV cells in the hippocampus [16, 48]. In the present study, while 2 weeks of venlafaxine or 2 weeks of EE do not modify the proportion of PV^+^ interneurons enveloped by PNN in CA1, the combination of venlafaxine and EE decreases the population of PV^+^/PNN^+^ cells in CORT depressed mice. From these data, it is tempting to speculate that PNN attenuation in CA1 may contribute to the rapid antidepressant action of venlafaxine combined with EE. It is not clear yet by what mechanisms PNN changes may translate into beneficial effects on depression symptoms. Although research on hippocampal function in depressed patients (or relevant animal models) is very scarce, studies have reported increased hippocampal activity in major depression, whereas antidepressants would counteract this effect by attenuating hippocampal activity [49, 50]. Since PNNs facilitate the firing of PV-expressing interneurons and probably the extracellular accumulation of GABA [51], their inactivation in response to the combination of venlafaxine and EE should, on the contrary, limit the tonic inhibition of hippocampal activity [12]. It is thus difficult to reconcile our data with this theory. Nevertheless, CORT can be considered to cause non-experience-dependent, or aberrant, PNN formation, resulting in strong inhibitory control of PV cells on the hippocampal circuit. In this context, degradation of PNN would overcome these constraints, and provide a time window during which appropriate experience-dependent plasticity on the PV cell network is possible.

To determine the mechanism by which changes in PNN can translate into beneficial effects on depression symptoms, we tested if preventing PNN degradation through the inhibition of metalloproteases (MMPs) could negatively reverberate on the rapid beneficial effects of venlafaxine associated with EE. Therefore, we used the tetracycline antibiotic doxycycline, a pharmacological agent crossing the blood-brain barrier [52], to inhibit MMPs activity [53]. We found that doxycycline prevents the ability of venlafaxine combined with EE to abolish emotional and cognitive symptoms of depression, after 2 weeks of treatment. Our results concur with the observation that the genetic or pharmacological inactivation of MMP9 decreases basal anxiety [54], despair and sociability in stressed animals [55] but also dampens the behavioral response of venlafaxine [15]. They are also consistent with data underlying the involvement of MMP polymorphisms in the development of depression [56, 57]. However, limitations related to the utilization of doxycycline in the diet have to be considered. Indeed, we cannot preclude that other molecular or cellular targets of the antibiotic doxycycline are involved in these behavioral effects. Since gut microbiota is known to influence emotional and cognitive processes [58], it is possible that slowing down the growth of specific bacteria played a negative role on emotionality. Other mechanisms may be involved, including the ability of doxycycline to reduce microglia activation [59, 60]. However, it seems unlikely that our data result from this property because the inactivation of microglia and neuroinflammation positively influences emotionality [61] and cognitive performances [62]. To better understand the link between MMP, PNNs and treatment response, it would now be interesting to examine the effects of a selective and potent inhibitor of MMP, notably a MMP9 inhibitor directly injected into the hippocampus, on PNN levels in our different experimental groups. Given the limitations of the use of doxycycline, we have implemented another approach. Indeed, since our results suggest that delayed antidepressant response might result from the formation of PNN in the hippocampus, we speculated that PNN digestion by chondroitinase (ChABC) might, instead, mimic the behavioral effects of venlafaxine combined with EE. We therefore sought to determine whether injecting ChABC into CA1 would elicit behavioral recovery. After showing that intra-hippocampal ChABC effectively reduced the presence of PNN locally, which likely results in a reduction of the inhibitory GABAergic tone [18, 51] leaving the hippocampus more excitable, we evaluated its behavioral outcomes. Remarkably, the sole administration of ChABC induced rapid neurobehavioral effects, as evidenced by its ability to improve the emotional and cognitive profiles of CORT-depressed mice. Interestingly, ChABC into CA1 also exerted an additive beneficial effect to venlafaxine on emotional state. This echoes recent data showing that the fast acting antidepressant ketamine also reduces the density of PNN [63]. This reinforces the idea that the modulation of hippocampal excitatory/inhibitory (E/I) balance by this extracellular matrix is crucial to reduce the delay of action of antidepressant drugs. Regarding the cognitive dimension, we found that the combination of ChABC and venlafaxine had no greater effect than ChABC alone. Although the reasons of different effects of ChABC on emotional and cognitive z-scores remain unknown, it seems unlikely that the injection of ChABC in CA1 has affected PNNs in CA2 since the social behavior of CORT-exposed mice was not altered [64] (Supplemental Fig. S4). Interestingly, a reduction of chondroitin sulfate proteoglycan, a major component of PNN, has been reported early after stress (i.e., 72 h), whereas an increase can be unraveled after 8 weeks. The latter finding underscores a pivotal role of the integrity of PV interneurons and their surrounding PNNs in mediating experience-dependent plasticity in the adult hippocampus [65–68].

In the search for the cellular and molecular mechanisms by which EE shortens the onset of action of antidepressants, our study did not explore hippocampal adult neurogenesis although there is mounting evidence that manipulating this process impacts hippocampal neuronal activity. In particular, stimulation of neurogenesis in the dentate gyrus, a process triggered by pharmacological [7, 21] and non-pharmacological antidepressant strategies [42, 69], has been shown to reduce CA1 neuronal activity [70]. In the present study we demonstrate that the combination of EE and venlafaxine reduces the PV^+^/PNN^+^ cell population in the CA1 region of CORT mice, while their number of PV^+^ cells in CA1 remains unchanged (Supplemental Fig. S2). From these data, we expect a decrease in inhibitory tone and thus an increase in neuronal activity in CA1 which is compatible with the results reported herein. This reinforces the interest in further exploring the relationship between PV+/PNN+ cell activity and adult hippocampal neurogenesis as an integrated mechanism that could underpin the onset of action of antidepressant drugs. More specifically, since dorsal and ventral hippocampus are functionally distinct structures (learning and emotions respectively associated with dorsal and ventral hippocampus) [71], it would be interesting in the future to study the ventral hippocampus. It is expected that the manipulation of PNN in this region will result in more pronounced effects on emotion and less robust effects on cognition.

## Supplementary information

Supplemental material
