# Supplementary material for: Enriched environmental exposure reduces the onset of action of the serotonin norepinephrin reuptake inhibitor venlafaxine through its effect on parvalbumin interneurons plasticity in mice
Source: Transl Psychiatry. 2023 Jun 26;13:227. doi: 10.1038/s41398-023-02519-x (PMC10293286; doi:10.1038/s41398-023-02519-x)
Supplement: Supplementary file 1 — Supplemental material [file 41398_2023_2519_MOESM1_ESM.docx]

**SUPPLEMENTAL DATA**

**Enriched environmental exposure reduces the onset of action of antidepressant through its effect on parvalbumin interneurons plasticity**

Basile Coutens, Camille Lejards, Guillaume Bouisset, Laure Verret, Claire Rampon^*^, Bruno Guiard^*^

Centre de Recherches sur la Cognition Animale (CRCA), Centre de Biologie Intégrative (CBI), CNRS UMR5169, Toulouse, France

**Materials and Methods**

***Elevated Plus Maze***

Mice were placed in the central platform (10 x 10 cm) of a maze consisting of a cross with two closed arms and two open arms (30 x 10 x 20 cm) elevated 50 cm from the ground. The time spent in closed and open arms was recorded for 5 minutes. Results were expressed as a percentage of time spent in the open arms.

***Object Location***

During the acquisition, two identical objects were placed in the middle of a squared arena (50cm) containing a visual cue (striped pattern). The mice could freely explore the environment for 10 min. On the next day, one object was moved, and the mice were allowed to explore for another 10 min.

***Tail suspension Test***

The tail suspension test evaluates the degree of resignation and is commonly used to screen antidepressant-like activity in mice [1]. Animals were suspended by the tail in a box containing a hook. During this test, the total immobility time was measured over 6 minutes.

***Three-chamber test***

Mice were placed in the center of a three-chamber setup (Moy et al., 2004), and were free to explore for 10min. Social recognition testing consisted of presenting a cage with a littermate (familiar) mouse, and another with an unfamiliar (new) mouse, never encountered before. Sociability test consisted of presenting a cage with a littermate (familiar) mouse compared to an empty cage. The investigation time for each cage was measured using the Ethovision software (Noldus).

***Splash Test***

The splash test was performed for 6 min as previously described [2]. After squirting 200 μL of a 10% saccharose solution on the back of the mice, latency of first grooming and grooming duration was scored manually as an index of self-care.

***Novelty Suppressed Feeding Test***

This test evaluates the aversion of rodents to eat in a stressful environment. The animals were placed under food restriction and weighed 24 hours before testing. Before the test, the animals were weighed again to ensure a weight loss of 10%. Then mice were placed in a squared arena containing food on a white filter paper located at the center of the arena under a bright light (~60 W) hanging about 60–80 cm above the food pellet. Latency to start eating was recorded with a cut-off time of 10 min. To rule out any hunger or motivation bias, each mouse was then placed in its homecage in the presence of a pellet for 5 minutes, during which latency to go to feed and food consumption were measured.

**
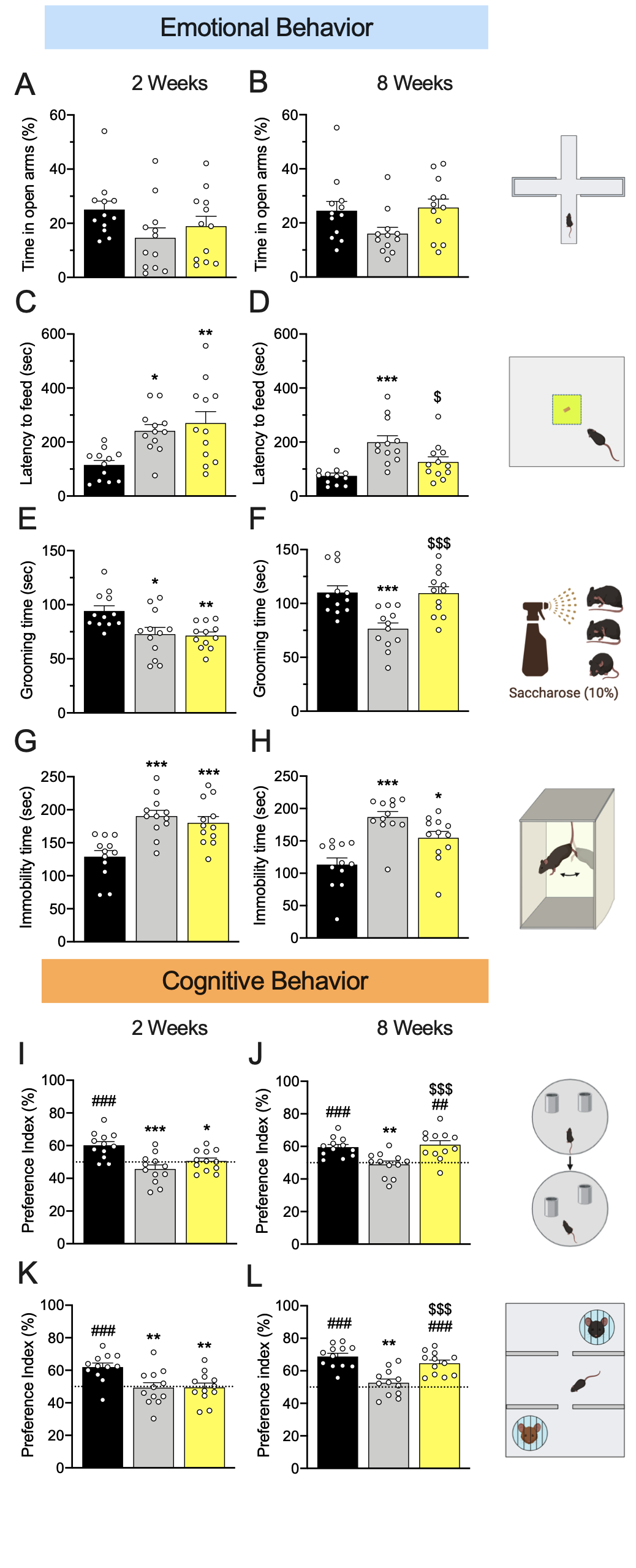
Figure S1**

**Supplemental Figure 1. Long-term treatment with venlafaxine is necessary to induce antidepressant effects in the CORT mouse model of depression.** Behavioral tests were performed after 2 and 8 weeks of treatment. One-way ANOVAs with treatment as main factor were applied for the two treatment durations. In the elevated plus maze no significant effect of treatment factor was detected after 2 and 8 weeks of treatment (A-B; F_(2;33)_=2.234 p=0.12 and F_(2;33)_=3.027 p=0.062, respectively). A significant effect of treatment factor was observed in the novelty suppressed feeding (C-D; F_(2;33)_=7.668, p=0.002 and F_(2;33)_=11.13, p=0.001, respectively), the splash test (E-F; F_(2;33)_=6.078, p=0.004 and F_(2,33)_=10.81, p=0.001, respectively), the tail suspension test (G-H; F_(2;33)_=12.55, p=0.001 and F_(2;33)_=14.32, p=0.001, respectively), the object location test (I-J; F_(2;33)_=10.80, p=0.001 and F_(2;33)_=9.530, p=0.001, respectively), and the social preference test (K-L; F_(2;33)_=6.949, p=0.003 and F_(2;33)_=15.94, p=0.001, respectively). Data represent mean ± SEM, dots illustrate individual values. Post-hoc analysis was conducted when appropriate: ^*^p < 0.05, ^**^p < 0.01, ^***^p < 0.001: significantly different from control (VEH) mice. ^$$$^p < 0.001: significantly different from CORT mice. ^###^p < 0.001: significantly different from chance level.

**Figure S2**

**
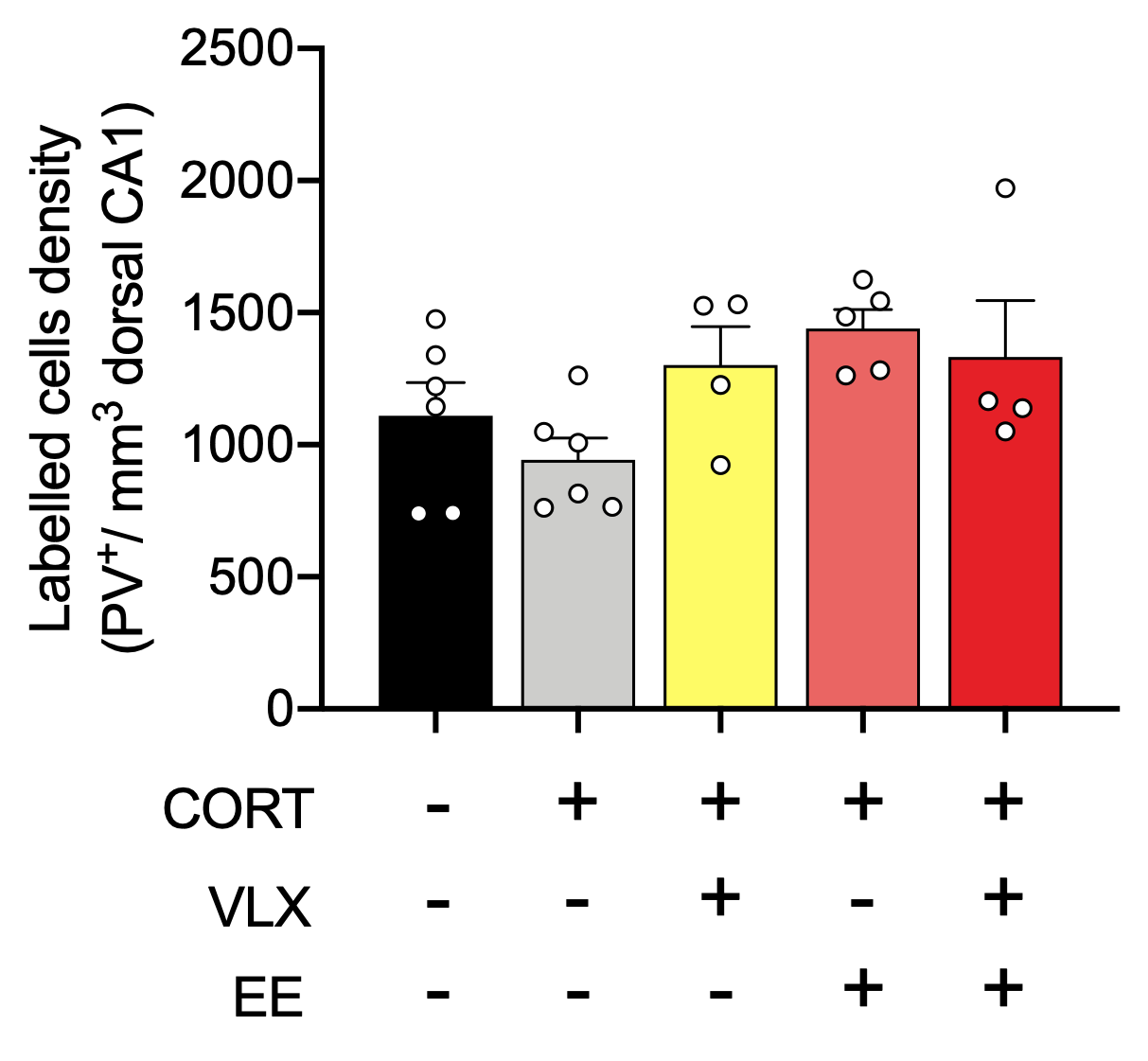
Therapeutic strategies did not change PV+ cell density into the dorsal CA1.** Density of PV^+^ cells in dorsal CA1. One-way ANOVA analysis did not reveal significant difference (F_(4;20)_=2.662, p = 0.062).

**Figure S3**

**
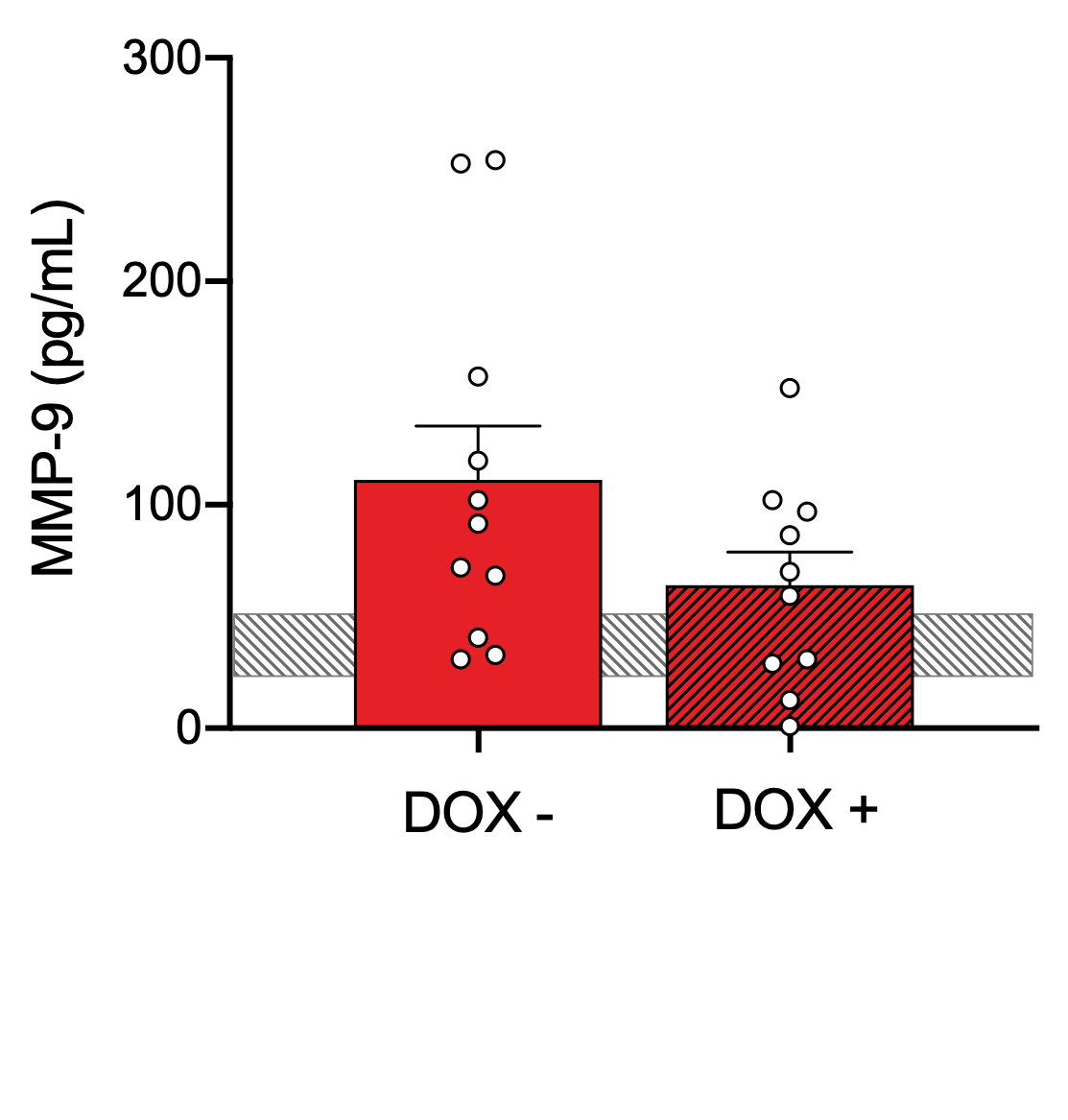
Effect of doxycycline on MMP-9 expression in whole hippocampus.** MMP-9 levels in hippocampal lysates. Doxycycline tend to decrease MMP-9 expression but statistical analysis did not revealed significant difference using unpaired Student t-test (t_(19)_=1.620 P=0.061)

**Figure S4**

**Pharmacological degradation of hippocampal PNN into the dorsal CA1 subregion of the hippocampus nor venlafaxine does not modify social behavior in CORT-exposed mice.** Index of preference for a familiar mice compared to an empty cage in the tree-chamber test. Behavioral test was realized in the same animal presented in Figure3 of the main manuscript. One simple t-test revealed that CORT-exposed mice do not present alteration of sociability. Moreover, One-way ANOVA do not reveled any difference between the different therapeutic strategies (F_(3;31)_= 0.7410, p= 0.53). # p < 0.05, ### p < 0.001 indicate significant differences compared to chance level (50%, dotted line).

**Table 1**

| **Experiments** | | **Statistical values from one-way ANOVA** | **Group comparison from post hoc tests** | **P values Tukey tests** | **Figures** |
| --- | --- | --- | --- | --- | --- |
| **EPM** | **2 weeks** | F_(2;33)_=2.23 P=0.123 | - VEH-STD vs. CORT-STD - VEH-STD vs. CORT-VLX - CORT-STD vs. CORT-VLX | P = 0.105  P = 0.438  P = 0.668 | FigS1A |
|  | **8 weeks** | F_(2;33)_=3.03 P=0.621 | - VEH-STD vs. CORT-STD - VEH-STD vs. CORT-VLX - CORT-STD vs. CORT-VLX | P = 0.133  P = 0.959  P = 0.077 | FigS1B |
| **NSF** | **2 weeks** | F_(2;33)_=7.668 P=0.002 | - VEH-STD vs. CORT-STD - VEH-STD vs. CORT-VLX - CORT-STD vs. CORT-VLX | P = 0.014  P = 0.002  P = 0.770 | FigS1C |
|  | **8 weeks** | F_(2;33)_=11.13 P<0.001 | - VEH-STD vs. CORT-STD - VEH-STD vs. CORT-VLX - CORT-STD vs. CORT-VLX | P < 0.001  P = 0.143  P = 0.025 | FigS1D |
| **Splash** | **2 weeks** | F_(2;33)_=6.708 P=0.004 | - VEH-STD vs. CORT-STD - VEH-STD vs. CORT-VLX - CORT-STD vs. CORT-VLX | P = 0.011  P = 0.007  P = 0.980 | FigS1E |
|  | **8 weeks** | F_(2;33)_=10.81 P<0.001 | - VEH-STD vs. CORT-STD - VEH-STD vs. CORT-VLX - CORT-STD vs. CORT-VLX | P < 0.001  P = 0.996  P = 0.001 | FigS1F |
| **TST** | **2 weeks** | F_(2;33)_=12.55 P<0.001 | - VEH-STD vs. CORT-STD - VEH-STD vs. CORT-VLX - CORT-STD vs. CORT-VLX | P < 0.001  P < 0.001  P = 0.717 | FigS1G |
|  | **8 weeks** | F_(2;33)_=14.32 P<0.001 | - VEH-STD vs. CORT-STD - VEH-STD vs. CORT-VLX - CORT-STD vs. CORT-VLX | P < 0.001  P = 0.014  P = 0.065 | FigS1H |
| **Object location** | **2 weeks** | F_(2;33)_=10.80 P<0.001 | - VEH-STD vs. CORT-STD - VEH-STD vs. CORT-VLX - CORT-STD vs. CORT-VLX | P < 0.001  P = 0.013  P = 0.281 | FigS1I |
|  | **8 weeks** | F_(2;33)_=9.53 P<0.001 | - VEH-STD vs. CORT-STD - VEH-STD vs. CORT-VLX - CORT-STD vs. CORT-VLX | P = 0.004  P = 0.871  P < 0.001 | FigS1J |
| **3-chambers test** | **2 weeks** | F_(2;33)_=6.949 P=0.003 | - VEH-STD vs. CORT-STD - VEH-STD vs. CORT-VLX - CORT-STD vs. CORT-VLX | P = 0.007  P = 0.008  P = 0.998 | FigS1K |
|  | **8 weeks** | F_(2;33)_=9.53 P<0.001 | - VEH-STD vs. CORT-STD - VEH-STD vs. CORT-VLX - CORT-STD vs. CORT-VLX | P = 0.004  P = 0.871  P < 0.001 | FigS1L |

**Table 2**

| **Experiment** | | **Statistical values from two-way ANOVA** | **Group comparison from post hoc tests** | **P values Tukey tests** | **Figures** |
| --- | --- | --- | --- | --- | --- |
| **Emotional Z-score** | **2 weeks** | Interaction: F_(1;59)_=4.16 P=0.0459  Treatment: F_(1;59)_=3.97 P=0.051  Housing condition: F_(1;59)_=27.0 P<0.001 | - CORT-VEH vs. CORT-VLX - CORT-VEH vs. CORT-EE - CORT-VEH vs. CORT-VLX-EE - CORT-VLX vs. CORT-EE - CORT-VLX vs. CORT-VLX-EE - CORT-EE vs. CORT-VLX-EE | N.A.  P = 0.146  P < 0.001  P = 0.137  P < 0.001  N.A. | Fig1B |
|  | **8 weeks** | Interaction: F_(1;59)_=5.57 P=0.023  Treatment: F_(1;59)_=14.9 P<0.001  Housing condition: F_(1;59)_=21.0 P<0.001 | - CORT-VEH vs. CORT-VLX - CORT-VEH vs. CORT-EE - CORT-VEH vs. CORT-VLX-EE - CORT-VLX vs. CORT-EE - CORT-VLX vs. CORT-VLX-EE - CORT-EE vs. CORT-VLX-EE | P < 0.001  P < 0.001  P < 0.001  P = 0.946  P = 0.459  P = 0.727 | Fig1C |
| **Cognitive Z-score** | **2 weeks** | Interaction: F_(1;59)_=1.84 P=0.179  Treatment: F_(1;59)_=0.024  P=0.878  Housing condition: F_(1;59)_=32.0 P<0.001 | - CORT-VEH vs. CORT-VLX - CORT-VEH vs. CORT-EE - CORT-VEH vs. CORT-VLX-EE - CORT-VLX vs. CORT-EE - CORT-VLX vs. CORT-VLX-EE - CORT-EE vs. CORT-VLX-EE | N.A.  P < 0.001  N.A.  N.A.  P = 0.013  N.A. | Fig1D |
|  | **8 weeks** | Interaction: F_(1;59)_=19.5 P<0.001  Treatment: F_(1;59)_=10.4 P=0.002  Housing condition: F_(1;59)_=8.39 P=0.006 | - CORT-VEH vs. CORT-VLX - CORT-VEH vs. CORT-EE - CORT-VEH vs. CORT-VLX-EE - CORT-VLX vs. CORT-EE - CORT-VLX vs. CORT-VLX-EE - CORT-EE vs. CORT-VLX-EE | P < 0.001  P < 0.001  P < 0.001  P = 0.994  P = 0.742  P = 0.841 | Fig1E |

**Table 3**

| **Experiments** | **Statistical values from one-way ANOVA** | **Group comparison from post hoc tests** | **P values Tukey tests** | **Figures** |
| --- | --- | --- | --- | --- |
| **Colocalization PV^+^/PNN^+^** | F_(4;20)_=6.378 P=0.002 | - VEH-STD vs. CORT-STD - VEH-STD vs. CORT-EE - VEH-STD vs. CORT-VLX - VEH-STD vs. CORT-EE-VLX - CORT-STD vs. CORT-EE - CORT-STD vs. CORT-VLX - CORT-STD vs. CORT-EE-VLX - CORT-EE vs. CORT- EE-VLX - CORT-VLX vs. CORT- EE-VLX | P = 0.002  P = 0.571  P = 0.125  P = 0.989  P = 0.064  P = 0.524  P = 0.013  P = 0.891  P = 0.362 | Fig2B |
|  | **Statistical values from Student t-test** |  |  |  |
| **Colocalization PV^+^/PNN^+^** | T_(14)_=8.200 | - CORT-VLX-EE vs. CORT- EE-VLX-DOX | P < 0.001 | Fig2F |
| **Emotional Z-score** | T_(19)_=4.464 | - CORT-VLX-EE vs. CORT- EE-VLX-DOX | P < 0.001 | Fig2G |
| **Cognitive Z-score** | T_(19)_=4.279 | - CORT-VLX-EE vs. CORT- EE-VLX-DOX | P < 0.001 | Fig2H |

**Table 4**

| **Experiment** | **Statistical values from one-way ANOVA** | **Group comparison from post hoc tests** | **P values Tukey tests** | **Figures** |
| --- | --- | --- | --- | --- |
| **Colocalization PV^+^/PNN^+^** | F_(3;31)_=11.00 P<0.001 | - CORT-VEH vs. CORT-ChABC - CORT-VEH vs. CORT-VLX - CORT-VEH vs. CORT-VLX-ChABC - CORT-ChABC vs. CORT-VLX - CORT-ChABC vs. CORT-VLX-ChABC - CORT-VLX vs. CORT-VLX-ChABC | P < 0.001  P = 0.599  P < 0.001  P = 0.023  P = 0.982  P = 0.009 | Fig3C |
|  | **Statistical values from two-way ANOVA** |  |  |  |
| **Emotional Z-score** | Interaction: F_(1;31)_=1.08 P=0.306  Pre-treatment: F_(1;31)_=9.06 P=0.0052  Treatment: F_(1;31)_=47.0 P<0.001 | - CORT-VEH vs. CORT-VLX - CORT-VEH vs. CORT-ChABC - CORT-VEH vs. CORT-VLX-ChABC - CORT-VLX vs. CORT-ChABC - CORT-VLX vs. CORT-VLX-ChABC - CORT-ChABC vs. CORT-VLX-ChABC | P = 0.526  P = 0.001  N.A.  N.A.  P < 0.001  P = 0.032 | Fig3D |
| **Cognitive Z-score** | Interaction: F_(1;31)_=00.0 P=0.985  Pre-treatment: F_(1;31)_=0.05  P=0.824  Treatment: F_(1;31)_=41.7 P<0.001 | - CORT-VEH vs. CORT-VLX - CORT-VEH vs. CORT-ChABC - CORT-VEH vs. CORT-VLX-ChABC - CORT-VLX vs. CORT-ChABC - CORT-VLX vs. CORT-VLX-ChABC - CORT-ChABC vs. CORT-VLX-ChABC | N.A.  P < 0.001  N.A.  N.A.  P < 0.001  N.A. | Fig3E |

Analyses of variance with one or two factors were used for the behavioral and immunohistochemical experiments. In case of homoscedasticity of the data (normality and homogeneity of variance) we used parametric tests. In case of heteroscedasticity of the data, we used non-parametric tests.

References

1. Steru L, Chermat R, Thierry B, Simon P. The tail suspension test: a new method for screening antidepressants in mice. Psychopharmacology (Berl). 1985;85:367–370.

2. David DJ, Samuels BA, Rainer Q, Wang J-W, Marsteller D, Mendez I, et al. Neurogenesis-dependent and -independent effects of fluoxetine in an animal model of anxiety/depression. Neuron. 2009;62:479–493.
